# Supplementary material for: Asynchronous magnetic resonance elastography: Shear wave speed reconstruction using noise correlation of incoherent waves
Source: Magn Reson Med. 2022 Oct 27;89(3):990–1001. doi: 10.1002/mrm.29502 (PMC9792433; doi:10.1002/mrm.29502)
Supplement: Supplementary file 1 — DATA S1 MATLAB code used to generate the numerical simulations of Figure 1 [file MRM-89-990-s002.zip › k-Wave/helpfiles/k-wave_release_notes.html]

Release Notes (k-Wave)


# Release Notes

**Current Version**: k-Wave Toolbox Version 1.3

## Contents

- V.1.3 (released 28 Feb 2020)
- V.1.2.1 (released 14 Dec 2017)
- V.1.2 (released 28 Aug 2017)
- V.1.1.1 (released 09 Oct 2015)
- V.1.1 (released 03 Oct 2014)
- V.1.0 (released 13 Nov 2012)
- B.0.5 (released 28 Feb 2012)
- B.0.4 (released 21 Dec 2011)
- B.0.3 (released 15 Feb 2011)
- B.0.2 (released 23 Jan 2010)
- B.0.1 (released 22 July 2009)

## Change Log - V.1.3

**Release Date:** 28 Feb 2020

**New Features and Changes:**

- axisymmetric simulations are now supported using `kspaceFirstOrderAS` and `kspaceFirstOrderASC`
- single-frequency simulations in homogeneous media can be computed without time-stepping using `acousticFieldPropagator` and `acousticFieldPropagatorC`
- time-varying sources in `kspaceFirstOrder1D`, `kspaceFirstOrder2D`, and `kspaceFirstOrder3D` (and the corresponding C++ codes) now include a k-space correction which improves accuracy for small numbers of points per period
- legacy behaviour for time-varying sources can be obtained by setting `source.p_mode = 'additive-no-correction'` and `source.u_mode = 'additive-no-correction'`
- holographic projections of planar input data can be performed using `angularSpectrum` and `angularSpectrumCW`
- measured pressure fields can be mapped to k-Wave inputs for accurate holographic projections using `calculateMassSource` and `calculateMassSourceCW`
- `'PMLSize'` can now be set to `'auto'` to automatically choose grid sizes with small prime factors
- 2D simulations can now be run using optimised C++ and C++/CUDA codes using `kspaceFirstOrder2DC` and `kspaceFirstOrder2DG`
- `kspaceFirstOrder3DC` (etc) now automatically calls the `combine_sensor_data` method if an object of the `kWaveTransducer` class is used as a sensor
- `kspaceFirstOrder3DC` (etc) now supports optional inputs to define the number of threads, thread binding, and the verbosity level
- the compiled C++ codes have been combined into two binaries now called `kspaceFirstOrder-OMP` (which supports 2D, 3D, and axisymmetric domains) and `kspaceFirstOrder-CUDA` (which supports 2D and 3D domains)
- the input file for the C++ code has been updated to version 1.2 (the k-space and shift variables, and PML vectors are no longer required)
- `pstdElastic2D` and `pstdElastic3D` can now return the particle velocity split into compressional and shear components by setting `sensor.record = {'u_split_field'}`
- `kWaveDiffusion` now allows command line updates to be suppressed by setting the optional input `'DisplayUpdates', false`
- `kWaveDiffusion` now prints a warning if dimension sizes have high prime factors
- `toneBurst` now allows the number of ring-up and ring-down cycles to be specified
- `makeDisc` and `makeBall` now allow non-integer radius values and maintain correct radius on axis
- `spect` and `extractAmpPhase` now work for 4D input matrices
- `waterAbsorption` now allows temperature to be defined as a vector
- `getWin` now allows `'Symmetry'` input to be defined as a vector
- `smooth` function inputs have changed (`kgrid` input no longer required)

**Bug Fixes:**

- bug fix in `waterSoundSpeed` for matrix inputs (generated error)
- bug fix in `focusedBowlONeil` when `axial_position = radius` (sometimes returned 0)
- bug fix in `getBLI` for functions with odd numbers of grid points (used incorrect wavenumbers)
- bug fix in `extractAmpPhase` for optional input `'Dim', 1` (generated error)
- bug fix in `kWaveDiffusion` when using the `plotTemp` method in 3D with an odd number of grid points (generated error)
- bug fix in `smooth` for matrices with even numbers of grid points (incorrectly filtered DC component)
- bug fix in Simulating B-mode Ultrasound Images and Simulating B-mode Images Using A Phased Array examples (attenuation value for time gain compensation given in incorrect units)

**New Functions:**

- `acousticFieldPropagator`
- `acousticFieldPropagatorC`
- `angularSpectrum`
- `angularSpectrumCW`
- `calculateMassSource`
- `calculateMassSourceCW`
- `getOptimalPMLSize`
- `kspaceFirstOrder2DC`
- `kspaceFirstOrder2DG`
- `kspaceFirstOrderAS`
- `kspaceFirstOrderASC`
- `reorderBinarySensorData`
- `roundEven`
- `roundOdd`
- `waterDensity`
- `waterNonlinearity`

**Deprecated Functions:**

- `speedSoundWater` (renamed to `waterSoundSpeed`)
- `attenuationWater` (renamed to `waterAbsorption`)

**New Examples:**

- Simulations In An Axisymmetric Coordinate System
- Simulating CW Fields Using The Acoustic Field Propagator
- Holographic Projections Using The Angular Spectrum Method
- Equivalent Source Holography

## Change Log - V.1.2.1

**Release Date:** 14th December 2017

**Bug Fixes:**

- bug fix in `kWaveDiffusion` when using `source.Q` with `medium.diffusion_coeff` (source term incorrectly scaled)

## Change Log - V.1.2

**Release Date:** 28 Aug 2017

**New Features and Changes:**

- thermal simulations are now supported using the `kWaveDiffusion` class and `bioheatExact`
- focused bowl transducers can be created using `makeArc` and `makeMultiArc` in 2D, and `makeBowl` and `makeMultiBowl` in 3D
- a warning is printed if dimension sizes have high prime factors
- default HDF5 compression level is now 0 (increases speed of saving input file for C++ codes)
- the compression level for HDF5 files can be set using the optional input `'HDFCompressionLevel'`
- movies are now saved using `VideoWriter` (see help for changes to optional inputs)
- `kspaceFirstOrder3DG` now allows the device number of the GPU to be selected
- `hounsfield2density` now returns matrices of the same data type as the input
- `kWaveGrid` is now implemented as a handle class (only handles are passed to functions to save memory)
- `kWaveGrid` now returns wavenumber vectors for discrete cosine and sine transforms
- `writeMatrix` now accepts an optional compression level input
- `makeGrid` has been deprecated, with grids created using the syntax `kgrid = kWaveGrid(...)`
- `makeTime` has been deprecated, and is now a method of `kWaveGrid`
- `makeTransducer` has been deprecated, with transducers created using the syntax `transducer = kWaveTransducer(...)`
- update to `overlayPlot` to allow optional inputs
- `scaleSI` now returns no scale for zero values
- `makeCircle` now supports circles not contained within the grid
- `speedSoundWater` now checks temperature limits
- update to style used in MATLAB documentation

**Bug Fixes:**

- bug fix in `kspaceFirstOrder1D`, `kspaceFirstOrder2D`, and `kspaceFirstOrder3D` for nonlinear simulations (convective nonlinearity term implemented incorrectly)- bug fix in using simulation functions in MATLAB 2016b and later (error with use of `nargin` and `nargout` in subscripts)
  - bug fix in `kspaceFirstOrder2D` when using `sensor.directivity_pattern = 'gradient'` (directional response incorrect)
  - bug fix in `kspaceFirstOrder3DG` when using a sensor mask defined by opposing corners and recording intensity (generated error)
  - bug fix in `kspaceFirstOrder3D-OMP` when using a `kWaveTransducer` source with checkpoint restart (wrong index used for time signal on restart)
  - bug fix in `kspaceLineRecon` and `kspacePlaneRecon` (incorrect use of `fftshift` caused energy loss at certain frequencies)
  - bug fix in `findClosest` (returned correct index but incorrect closest value)
  - bug fix in setting apodization in `kWaveTransducer` class with only one active element (returned NaN)
  - bug fix in `writeGrid` for odd dimension sizes (used even wavenumber components)

**New Functions:**

- `bioheatExact`
- `createCWSignals`
- `extractAmpPhase`
- `focusedBowlONeil`
- `fourierShift`
- `getComputerInfo`
- `kWaveDiffusion`
- `makeArc`
- `makeMultiArc`
- `makeBowl`
- `makeMultiBowl`

**Deprecated Functions:**

- `makeGrid`
- `makeTime`
- `makeTransducer`
- `timeShift`

**New Examples:**

- Heat Diffusion In A Homogeneous Medium
- Constant Rate Of Heat Deposition
- Using A Binary Sensor Mask
- Heating By A Focused Ultrasound Transducer
- Iterative Image Reconstruction Using The Adjoint

## Change Log - V.1.1.1

**Release Date:** 9th October 2015

**Bug Fixes:**

- bug fix in `pstdElastic2D` and `pstdElastic3D` when defining material coefficients on staggered grid (generated incorrect results)

## Change Log - V.1.1

**Release Date:** 3rd October 2014

**New Features and Changes:**

- simulations in elastic media are now supported using the `pstdElastic2D` and `pstdElastic3D` functions
- 3D simulations can be run on an NVIDIA graphics processing unit (GPU) using a native C++/CUDA code
- rectangular binary sensor masks can be defined by assigning the grid coordinates of two opposing corners to `sensor.mask`
- `sensor.record` now supports the additional input options `'p_min'`, `'p_max_all'`, `'p_min_all'`, `'u_min'`, `'u_max_all'`, `'u_min_all'`, and `'u_non_staggered'`
- simulation functions have been restructured to reduce code duplication
- the accuracy of the acoustic intensity output for frequencies close to Nyquist limit has been improved
- the C++ code now supports command line inputs for checkpoint-restart and importing FFTW wisdom
- the C++ code now exploits AVX instructions in addition to SSE
- attenuation compensation for photoacoustic tomography can be performed using time-variant filtering via `attenComp`
- vessel filtering for photoacoustic tomography can be performed using `vesselFilter`
- `kspaceFirstOrder3DC` now supports optional inputs to specify the name and location of the binary and data files
- `makeSphere` and `makeSphericalSection` can be set to return logical matrices to reduce memory usage
- `resize` now supports 1D inputs
- `checkFactors` now also reports prime numbers

**Bug Fixes:**

- bug fix in using a Cartesian sensor mask with nearest neighbour interpolation and `sensor.record` set to `'p_min'` (generated error)
- bug fix in using `sensor.directivity_angle` with the optional input `'DataCast'` set to `'gpuArray-single'` (generated error)
- bug fix in `kspaceFirstOrder3DC` when performing time reversal image reconstruction using `sensor.time_reversal_boundary_data` with `'PMLInside'` set to `false` (PML was not removed)
- bug fix in `kspaceFirstOrder3DC` when calling script on a different drive using Windows (generated error)
- bug fix in `kspaceSecondOrder` when setting absorption values with `medium.alpha_power` to values less than 1 (returned NaN)
- bug fix in `kspaceSecondOrder` when using `sensor.record = {'p_final'}` (generated error, grid expansion not removed)
- bug fix in `makeSphere` for grid sizes with uneven dimensions (generated error)
- bug fix in `makeSphere` to centre sphere on the kgrid origin (previously offset by 1)
- bug fix in `writeMatrix` for source variables larger than 1 MB (incorrect chunk size generated error)

**New Functions:**

- `attenComp`
- `checkStability`
- `fitPowerLawParams`
- `getBLI`
- `getSpacedPoints`
- `h5compare`
- `kspaceFirstOrder3DG`
- `maxND`
- `minND`
- `overlayPlot`
- `pstdElastic2D`
- `pstdElastic3D`
- `revolve2D`
- `timeShift`
- `vesselFilter`
- `writeAttributes`
- `writeFlags`
- `writeGrid`

**New Examples:**

- Defining A Sensor Mask By Opposing Corners
- Attenuation Compensation Using Time Variant Filtering
- Running C++ Simulations
- Saving Input Files in Parts
- Explosive Source In A Layered Medium
- Plane Wave Absorption
- Shear Waves And Critical Angle Reflection
- Simulations In Three Dimensions

## Change Log - V.1.0

**Release Date:** 13th November 2012

**New Features and Changes:**

- 3D simulations can now be run using an optimised C++ code
- the data recorded by the sensor mask is now set using `sensor.record = {'p', 'u', 'p_final', ...}`
- time-varying and averaged acoustic intensity can be returned by setting `sensor.record = {'I', 'I_avg', ...}`
- the inputs `'ReturnVelocity'` and `sensor.record_mode` and the output `field_data` have been deprecated
- the time index at which the sensor starts recording can be set using `sensor.record_start_index`
- `'DataCast'` can now be set to `'gpuArray-single'` or `'gpuArray-double'` to automatically run simulations on a graphics processing unit (GPU) using the Parallel Computing Toolbox (R2012a or later)
- output variables are now created and returned using the data type specified by `'DataCast'`
- output variables can be returned in double precision by setting `'DataRecast'` to `true`
- Cartesian sensor masks with linear interpolation are now supported for all `'DataCast'` settings and the interpolation speed during runtime has been significantly improved
- `'CartInterp'` set to `'linear'` is now the default setting for 1D, 2D, and 3D simulations
- the calculation of `kgrid.k` within `makeGrid` has been modified to improve memory efficiency
- `spectrum` has been renamed to `spect` to avoid a naming conflict with the MATLAB signal processing toolbox, and now accepts matrix inputs
- `makeTime` now uses the maximum sound speed to compute `dt`, and the minimum sound speed to compute `t_end`
- `unmaskSensorData` function inputs have changed

**Bug Fixes:**

- bug fix in using `'PlotLayout'` set to `true` when `medium.sound_speed` or `medium.density` are scalar (generated plot error)
- bug fix in list of data cast variables for `'DataCast'` set to `'GPUsingle'`
- bug fix in setting the source scale parameter for time varying pressure sources in heterogeneous media with `p_mode` set to `'dirichlet'`
- bug fix in setting the axis limits in `kspaceFirstOrder2D` with `'MeshPlot'` set to `true`
- bug fix in `makeGrid` when defining the wavenumber variables `kgrid.k`, `kgrid.kx` (etc) for grid sizes with an odd number of grid points
- bug fix in `makeSphere` for grid sizes with an odd number of grid points
- bug fix in `getWin` for `type` set to `'Gaussian'` or `'Kaiser'` with a user input for `'Param'` (param value was not used)
- bug fix in the `scan_line` method of `kWaveTransducer` for scan lines formed using a negative steering angle (generated error)
- bug fix in `grid2cart` for 1D inputs (list of Cartesian points returned in the wrong direction)
- bug fix in computing axis units in `kspaceSecondOrder`

**New Functions:**

- `beamPlot`
- `focus`
- `getFDMatrix`
- `gradientSpect`
- `gradientFD`
- `kspaceFirstOrder3DC`
- `makeLine`
- `makeSphericalSection`
- `reorderSensorData`
- `scaleFig`
- `scanConversion`
- `writeMatrix`

**New Examples:**

- Simulating B-mode Images Using A Phased Array

## Change Log - B.0.5

**Release Date:** 28th February 2012

**New Features and Changes:**

- time varying pressure and velocity inputs can now be either applied as mass and force sources (the default), or enforced as a Dirichlet boundary condition by setting `source.p_mode` and `source.u_mode` to `'dirichlet'`
- the implementation of source terms in `kspaceFirstOrder1D`, `kspaceFirstOrder2D`, `kspaceFirstOrder3D` has been modified to reduce code duplication
- the performance of the PML has been improved and the default value for `'PMLAlpha'` changed to `2`
- the plotting speed in `kspaceFirstOrder1D` has been improved
- the axis limits for the `'PlotLayout'` option have been modified to allow easier interpretation
- the default status bar position has been shifted to below the plot window
- estimated simulation times are now based on the first 50 times steps with the first step excluded
- `stackedPlot` functionality has been extended and the default axis limits fixed
- help files are now searchable using the MATLAB Help browser and k-Wave version is listed using `ver`

**Bug Fixes:**

- bug fix in setting the scale parameter for time varying pressure and velocity sources in heterogeneous media
- bug fix in setting the scale parameter using the `kWaveTransducer` class (no scaling was applied)
- bug fix in applying long time varying sources using the `kWaveTransducer` class (data type was set incorrectly)
- bug fix in `kspaceFirstOrder1D` using velocity sources (source incorrectly named)
- bug fix in using `'PlotLayout', true` with `'DataCast', 'gsingle'` (generated blank jacket figure)
- bug fix in `'StreamToDisk'` option (error generated if `kgrid.Nt` exactly divisible by saving frequency)

**New Functions:**

- `hounsfield2density`
- `sharpness`

**New Examples:**

- 2D Iterative Image Improvement Using Time Reversal
- Automatic Sound Speed Selection

## Change Log - B.0.4

**Release Date:** 21st December 2011

**New Features and Changes:**

- the indexing of input matrices has been modified to (x, 1) in 1D, (x, y) in 2D and (x, y, z) in 3D to be more intuitive
- the corresponding inputs to `makeGrid` are now (Nx, dx) in 1D, (Nx, dx, Ny, dy) in 2D, and (Nx, dx, Ny, dy, Nz, dz) in 3D- cumulative nonlinear effects can be modelled by setting `medium.BonA`
  - heterogeneous absorption values can now be used by defining `medium.alpha_coeff` as a matrix
  - `kspaceFirstOrder1D`, `kspaceFirstOrder2D`, `kspaceFirstOrder3D`, and `makeGrid` have been re-written to significantly reduce memory usage
  - `source` and `sensor` inputs can be defined as an object of `kWaveTransducer` type (returned by `makeTransducer`) to allow simple simulations using ultrasound transducers
  - `source.ux`, `source.uy`, and `source.uz` can be specified individually
  - time varying inputs `source.p`, `source.ux`, etc., can now contain any number of time points (they do not need to be the same length as `kgrid.t_array`)- approximate GPU memory usage is reported when using k-Wave with the GPUmat or Accelereyes toolboxes
    - the reference sound speed used in the k-space operator can now be manually set by defining `medium.sound_speed_ref`
    - the statistics of the pressure field at each sensor element can now be computed without storing the pressure field by setting `sensor.record_mode` to `'statistics'`- 3D `sensor_data` can now optionally be streamed to disk by setting the optional input parameter `'StreamToDisk'` to `true`
      - `toneBurst` function inputs have changed
      - `db2neper` and `neper2db` now use exact conversion factor
      - frequency filtering applied when `sensor.frequency_response` is given is now performed using `gaussianFilter`
      - B.01 release style inputs no longer supported

**Bug Fixes:**

- bug fix in defining the absorption parameters for heterogeneous media
- bug fix in setting initial velocity for `kspaceFirstOrder3D`
- bug fix in frequency filtering applied when `kgrid.t_array` has an odd number of time points
- bug fix in `kspaceFirstOrder2D` when saving movies with `'MovieType'` set to `'image'` (the first frame did not display the sensor mask), and the movie frames variable is now preallocated
- bug fix in creating the spatial wavenumber matrices using the `makeGrid` class when 1/Nx (etc) is a recurring number

**New Functions:**

- `checkFactors`
- `envelopeDetection`
- `findClosest`
- `gaussianFilter`
- `logCompression`
- `makeTransducer`
- `mendousse`
- `stackedPlot`

**New Examples:**

- Defining An Ultrasound Transducer
- Simulating Ultrasound Beam Patterns
- Using An Ultrasound Transducer As A Sensor
- Simulating B-mode Ultrasound Images
- Modelling Nonlinear Wave Propagation

## Change Log - B.0.3

**Release Date:** 15th February 2011

**New Features and Changes:**

- support for time varying velocity inputs via `source.u_mask`, `source.ux` etc
- sensor data now also returns velocity if `'ReturnVelocity'` is set to `true`
- sensor frequency response can be defined using `sensor.frequency_response`
- time reversal image reconstructions can include compensation for acoustic attenuation
- `'CartInterp'` set to `'linear'` now uses `TriScattererdInterp` and is supported in 3D
- `'PMLInside'` set to `false` is now supported in 3D
- estimated simulation times are given
- the PML can be hidden by setting `'PlotPML'` to `false`
- `'PlotScale'` can now also be set to `'auto'` and the display can be log compressed by setting `'LogScale'` to `true`
- 2D simulations can now also be displayed as a mesh plot by setting `'MeshPlot'` to `true`
- movie frame capture in 2D can be controlled using `'MovieType'`
- default smoothing now only applies to the initial pressure distribution
- `kgrid.x_off` (etc) fields are no longer supported
- bug fix in setting initial velocity for time staggered grids
- new k-space simulation function `kspaceSecondOrder` based on a second-order lossy wave equation for homogeneous media
- `makeGrid` now uses class definitions
- `spectrum` now supports all window shapes defined in `getWin`
- `smooth` now uses a Blackman window as default and supports all window shapes defined in `getWin`
- `resize` function inputs have changed and now supports 3D inputs
- bug fix in `kspacePlaneRecon` remapping

**New Functions:**

- `addNoise`
- `applyFilter`
- `benchmark`
- `expandMatrix`
- `fwhm`
- `gaussian`
- `getAlphaFilter`
- `getDateString`
- `getWin`
- `interpftn`
- `kspaceSecondOrder`
- `saveTiffStack`
- `voxelPlot`

**New Examples:**

- Recording The Particle Velocity
- Defining A Gaussian Sensor Frequency Response
- Comparison Of Modelling Functions
- Setting An Initial Pressure Gradient
- Photoacoustic Waveforms in 1D, 2D and 3D
- Dipole Point Source In A Homogeneous Propagation Medium
- The Doppler Effect
- Diffraction Through A Slit
- Focussed Detector in 3D
- Modelling Sensor Directivity in 2D
- Modelling Sensor Directivity in 3D
- Focussed 2D Array with Directional Elements
- Image Reconstruction With Directional Sensors
- Image Reconstruction With Bandlimited Sensors
- Image Reconstruction With Compensation For Acoustic Absorption
- Source Smoothing
- Modelling Power Law Absorption

## Change Log - B.0.2

**Release Date:** 23rd January 2010

**New Features:**

- inputs redefined as structures
- power law absorption
- 2D sensor directivity
- time varying pressure inputs

**New Functions:**

- `attenuationWater`
- `db2neper`
- `filterTimeSeries`
- `grid2cart`
- `neper2db`
- `numDim`
- `powerLawKramersKronig`
- `scaleSI`
- `scaleTime`
- `spectrum`
- `speedSoundWater`
- `toneBurst`

## Change Log - B.0.1

**Release Date:** 22nd July 2009

First Beta Release.
